# High but decreasing prevalence of overweight in preschool children: encouragement for further action

**DOI:** 10.1136/bmj-2023-075736

**Published:** 2023-10-10

**Authors:** Sarah E Maessen, Melanie Nichols, Wayne Cutfield, Shane A Norris, Christoph Beger, Ken K Ong

**Affiliations:** 1 Better Start National Science Challenge, Auckland, New Zealand; 2 Department of Paediatrics: Child and Youth Health, University of Auckland, New Zealand; 3 Global Centre for Preventive Health and Nutrition, Institute for Health Transformation, Deakin University, Geelong, Australia; 4 Liggins Institute, University of Auckland, New Zealand; 5 SAMRC/Wits Developmental Pathways for Health Research Unit, Faculty of Health Sciences, University of the Witwatersrand, Johannesburg, South Africa; 6 School of Human Development and Health, University of Southampton, UK; 7 LIFE Child and CrescNet, Center for Pediatric Research, Leipzig University Hospital for Children and Adolescents, Leipzig University, Leipzig, Germany; 8 MRC Epidemiology Unit, Institute of Metabolic Science, and Department of Paediatrics, University of Cambridge, UK

## Abstract

**Sarah Maessen and colleagues** argue that identifying successful policies and practices in countries with falls in early childhood overweight can help enhance efforts and reduce within country inequities

Nearly every country worldwide has experienced a steady rise in the prevalence of obesity across all age groups in recent decades.^1^ However, there has been recent optimism about an apparent plateau in prevalence of unhealthy weight among children in some high income countries, particularly among young children.^2^ A World Health Organization analysis of national surveys from 144 countries described notable increases in the prevalence of overweight and obesity among preschool children (under 5 years) from 1990 to 2010, and predicted an acceleration in prevalence from 2010 to 2020.^3^ WHO highlighted the preschool years as a potentially critical time in the life course for development of obesity and related metabolic disorders, with most preschool children with overweight or obesity continuing to be above a healthy weight as older children and adults.^4^ We analyse structural and policy changes in countries that do not seem to be experiencing the predicted rises in prevalence of overweight and obesity among preschool children and urge policy makers to build on this progress and ensure benefit across all societal groups.

## Countries with stable or declining trends 

Data on trends in overweight and obesity prevalence are scarce for young children and, where available, show considerable heterogeneity between and within countries. We examined national or regionally representative data on trends in early childhood body mass index (BMI) from five countries, four of which were high income (box 1). To support robust temporal and geographical comparisons, we used a threshold of >1 standard deviation above average weight across settings to define overweight, roughly equivalent to the 85th percentile.^4^ Although more extreme thresholds are used to define obesity in clinical practice, less stringent thresholds are more useful for population level comparisons.

Box 1 **Data on prevalence of overweight in young children**

- New Zealand: near universal national data from the routine age 4-5 years B4 school check^5^
- Victoria, Australia: state-wide data collected by the universal maternal and child health service, from routine clinic visits at age 3.5 years^6^
- Germany: nationally representative data on young children attending routine medical checks collected through the CrescNet database that connects 247 paediatricians throughout the country. Data on children aged 4-6 years were summarised.^7^
- England: the National Childhood Measurement Programme is a mandated annual programme of measurements in schools at ages 4-5 and 10-11 years.^8^
- South Africa: national or provincial data on the prevalence of overweight in young children using differing survey designs, including the National Food Consumption Survey (1999, 2005), the South African National Health and Nutrition Examination Survey (2012), and the Provincial Dietary Intake Study (2018).^9^

**Fig 1:**
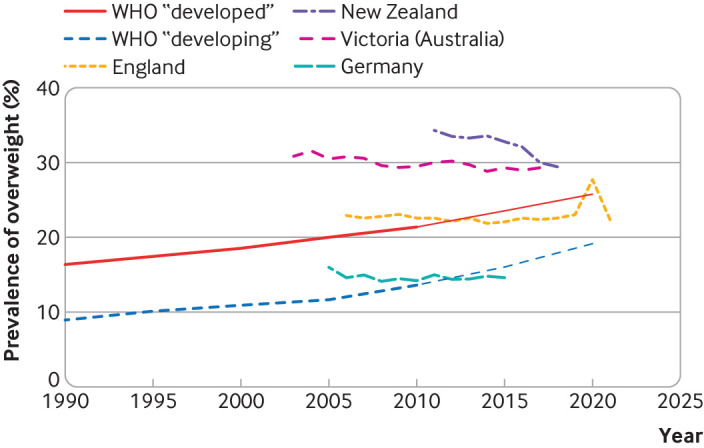
Trends in the prevalence of overweight in young children. Data show WHO estimates among countries that it termed as “developed” or “developing” 1990 to 2010 and predicted values for 2015 to 2020 (extrapolated from WHO predicted values for weight:height >2 SD above average)^4^ National or regional lines show overweight defined as weight:height or BMI >1 SD (equivalent to the 85th percentile). Note, for young children WHO termed this threshold “at risk of overweight” and based its analyses on weight:height values but reported similar prevalences when it applied this threshold to BMI values^3^

The prevalence of overweight in preschool children fell by around 15% in New Zealand between 2010-11 and 2018-19,^5^ around 5% in the Australian state of Victoria between 2003 and 2017,^6^ and around 9% in Germany between 2005 and 2015^7^ (fig 1). In England, the prevalence of overweight has been mostly stable among 4-5 year old children, with a gradual relative decrease of around 3% between 2006-07 and 2021-22. This contrasts with a steady increase in prevalence of overweight and obesity observed in children aged 10-11 in England.^8^ However, in both age groups the prevalence of overweight increased sharply during the covid-19 pandemic in 2020-21, with a relative increase of around 20% in 4-5 year olds.

Although comprehensive data are lacking from lower income countries, data from South Africa,^9^ a nutritionally transitioning upper middle income country, show a substantial rise in early childhood overweight from 19-20% in 1999-2005 to 38-41% in 2012-18, exceeding the rises predicted by WHO^3^.

## Potential underlying drivers

**Fig 2:**
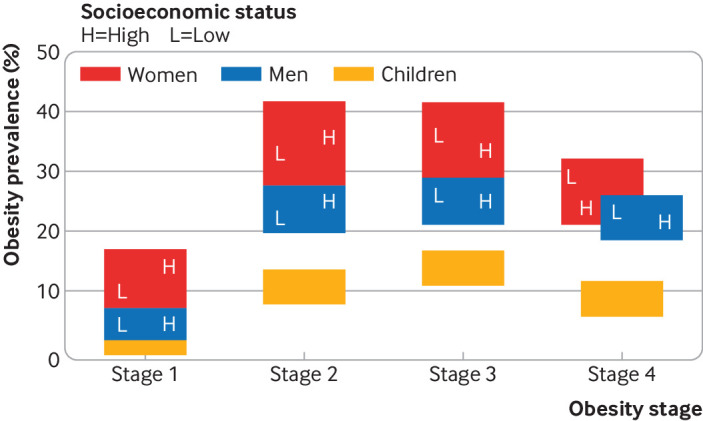
Four stages of the obesity epidemic. The distribution of obesity between socioeconomic groups changes as countries progress through the stages. Progress is related to economic development and local factors. The fourth stage remains putative as too few countries have reversed increases in obesity. Adapted from Jaacks et al^1^

The patterns of early childhood overweight in high income countries fit the fourth, and final, stage of a conceptual model for transition in obesity^1^ with plateauing or decreasing trends in overweight and obesity, albeit remaining at very high levels (fig 2). However, it is challenging to pin down the specific reasons for the stabilisation and decline in prevalence among young children in these settings. The determinants of overweight and obesity are complex, spanning individual behaviour, genetic factors, culture, and environmental drivers. Various structural factors may be driving these population level trends, encompassing environmental, social, policy, and commercial determinants of diet and physical activity (box 2).

Box 2 **Structural determinants of healthy weight in young children**

- Environment**—**eg, space and facilities for outdoor play; infrastructure for active travel to school; density of takeaway outlets; accessibility to fresh food stores
- Social—eg, awareness of early childhood overweight and overfeeding; maternal smoking in pregnancy
- Policy—eg, provision of early years education and childcare; provision and promotion of healthy food and physical activity in early education settings; appropriate growth references for weight monitoring and parental feedback
- Commercial—eg, reformulation of foods and drinks to reduce free sugars; reduction in protein content of infant milk formulas

Young children may be the first age group to show a plateau or decline in overweight because preventive efforts may be more effective when begun earlier in life^4^ and because parents of infants and young children have repeated contacts with health professionals and tend to be receptive to their advice on feeding and caring. Attention has been increasing over the past two decades on preventing early obesity, with more than 60 ongoing or completed trials worldwide.^10^ Furthermore, many countries have adopted the WHO 2006 International Growth Standards, which describe optimal growth rather than population norms and classify more 0-5 year olds as overweight than previous growth charts.^11^ Many countries have programmes for monitoring growth in infants and preschool children and feedback on overweight status facilitates access to referral and support pathways and encourages some parents to seek further information.^12^ Although evidence of subsequent lifestyle changes is limited, no unfavourable effects have been detected.^12^

Childcare arrangements show complex associations with overweight and obesity, with parental care typically conferring the lowest risk, especially when compared with informal non-parental care, such as by grandparents.^13^ The introduction of six weeks’ paid parental leave in California in 2002 was associated with a 4.1% relative reduction in childhood overweight at school entry with a larger 14.7% reduction seen in children of mothers with low educational attainment. These trends may be related to increased breastfeeding, prompt infant health check-ups, lower prenatal stress, and less non-parental infant care.^14^ New Zealand introduced paid parental leave in 2002, with availability increasing from 2004 to 2020, and it was introduced in Australia in 2010. 

In addition, governments have made substantial investments in formal early childhood education and care. New Zealand introduced a policy to provide 20 hours of free early childhood education a week for 3-5 year olds alongside a series of reforms throughout the 2000s increasing the number of facilities and enrolments, teachers’ qualifications, and the affordability of childcare.^2^ England introduced 15 hours of free childcare a week for all children aged 3 and 4 years in 2010; this was increased to 30 hours a week in 2017, and the 15 hours offer was extended to 2 year olds from disadvantaged families in 2013. In Germany, the Day Care Expansion Act regulated a demand oriented expansion of education and childcare provision for children under 3, and uptake has grown steadily since its implementation in 2005.^15^ Importantly, these investments have been accompanied by policies and curriculum changes to promote healthy food and physical activity in educational and daycare environments. For example, in New Zealand, centres must promote or provide food that meets healthy eating guidelines, with targeted funding available for centres serving less affluent families. In Australia, over the past 10 to 15 years there has been a shift to greater use of daycare centres, which typically provide all food and drinks. National quality standards and regulations mandate minimum nutrition standards and environments that promote physical activity.

Maternal smoking is associated with a 1.50 higher risk of obesity at age 4,^16^ possibly because of the interlinked effects on lower birthweight, more rapid postnatal weight gain, and reduced breastfeeding as well as the independent effect of secondhand smoke exposure in early life.^17^ Substantial reductions in the prevalence of maternal smoking in pregnancy over recent years indicate progress in public health and maternal and child health services in high income countries. In England, prevalence of maternal smoking at end of pregnancy reduced from 15.8% to 9.1% between 2006-07 and 2021-22; in Australia smoking at any time during pregnancy reduced from 13.7% to 9.2% between 2010 and 2020; and in New Zealand smoking at first antenatal visit reduced from 16.2% to 13.1% between 2006 and 2018.

Regarding diet and nutrition, any breastfeeding and longer breastfeeding duration are both consistently associated with lower risk of childhood obesity.^18^ Although debate remains about whether this association is fully causal, the overall prevalence of any breastfeeding in high income countries increased between 2000 and 2019.^19^ Furthermore, widespread changes in infant formula composition have reduced protein levels, a determinant of rapid infant weight gain and early childhood BMI.^20^ Beyond infancy, potential dietary targets to prevent overweight in young children include reducing portion sizes of snacks and meals in preschool settings and reducing intakes of protein and sugar sweetened beverages.^21^ Sales of sugary drinks have fallen greatly in high income countries over the past two decades, with larger reductions in countries that introduced fiscal policies, such as the UK’s soft drinks industry levy. However, data are limited on trends in consumption in preschool children.^22^

We may also learn about how to reduce childhood overweight from knowledge of the factors that have increased it. Several factors have been proposed to explain the increases in overweight during the covid-19 pandemic. Although increases were seen across all age groups, children were particularly affected by school and childcare closures, with greater increases in sedentary time (by around 160 min/day) than seen in adults,^23^ and increased mental health problems but with mixed effects on dietary patterns (more sweets and snacks but fewer takeaways).^24^ While the reduction in prevalence of early childhood overweight to pre-pandemic levels in 2021-22 in England is reassuring (fig 1), these large differences indicate sensitivity of weight to changes in structural factors.

Levels of overweight and obesity have increased rapidly across all age groups in South Africa.^9^ Despite the adoption of WHO 2006 International Growth Standards and the WHO Nurturing Care framework into the country’s *Road to Health* booklet for postnatal care, there has been little improvement in early risk factors for overweight. South Africa has one of the lowest breastfeeding rates on the African continent and has not seen the reductions in maternal smoking reported in high income countries; indeed, the greatest uptake of tobacco use is among younger women. Furthermore, South African culture does not typically perceive childhood overweight or obesity as a concern; rather, typical attitudes prefer a chubbier baby.^25^ Therefore, alongside the structural changes and policies described above, interventions are needed to change unhelpful cultural norms around feeding—for example, through education and engagement to harness the strong matriarchal influence and through use of peer counsellors.^25^

## Vulnerable groups

Although overall levels of overweight in young children are reducing, there is significant heterogeneity between population subgroups. For example, in the UK, the prevalence of early childhood obesity increased from 12.2% to 13.6% among the most deprived decile between 2006-07 and 2021-22.^8^ UK data also show that although the overall prevalence of overweight fell among 4-5 year-olds during this period, the prevalence of severe obesity (BMI >99.6th percentile) increased from 2.4% to 2.9%.^8^ In Victoria, Australia, state-wide decreases in early childhood overweight were seen across all socioeconomic groups between 2003 to 2017; however in non-metropolitan areas, increasing trends in overweight and mean BMI z-score were consistently seen in all age and sex groups and across socioeconomic groups.^6^ These geographical differences might reflect inequities in the accessibility of health services and early childhood care and education.

To our knowledge, New Zealand is the only setting where disparities in overweight across sex, ethnicity, and affluence subgroups seem to be narrowing.^5^ This may be the result of the government’s promotion of child welfare in general throughout the 1990s and 2000s, alongside increased health literacy and understanding of a healthy diet for optimal growth and development. In addition, maternal smoking rates in specific population subgroups in New Zealand (including the Māori and Pasifika communities, and less affluent groups) were very high but have fallen steeply in recent years; this may have contributed to the decline in early overweight prevalence. However, as living costs continue to rise for New Zealand families as well as globally, successful policies need to be reviewed and enhanced to ensure these gains are not lost.

## Building on progress

Changes in the prevalence of early childhood overweight may reflect the level of influence of structural factors, including environmental, social, policy, and commercial determinants of eating patterns, diet, physical activity, and sedentary time in individual children and families. Observed plateaus or declines in Australia, Germany, New Zealand, and UK, albeit still at high levels, should provide encouragement that further structural interventions are likely to continue and hasten these declines. Proposed future policy tools include regulation of marketing of unhealthy food and drinks to young children, greater support for breastfeeding and healthy infant feeding, investment in the built environment to facilitate active children, and ensuring that schools and nurseries play a central role.^26^ Policy changes should be robustly evaluated, and additional focus is needed to avoid increasing inequities in levels of early childhood overweight as well as to maintain these improvements into later childhood and adolescence. Furthermore, additional evidence is needed to inform policies in low and middle income countries where the prevalence of early childhood overweight continues to rise.

**Key messages**

- Levels of overweight and obesity among preschool children are stable or declining in several high income countries
- Possible contributing factors include increasing attention on early obesity prevention, changes in infant feeding, investments in early childhood education and childcare, and reduced levels of maternal smoking
- Despite reductions, levels of early childhood overweight remain high and have increased in some vulnerable groups and during particular periods, such as the covid-19 pandemic
- These trends highlight the sensitivity of preschool overweight and obesity to structural factors and the need for further action to hasten and extend weight reductions

## Notes

Contributors and sources: KKO conceived the idea. SEM, MN, WC, and KKO were involved in the conceptualisation of the analysis. SEM, MN, SAN, CB, and KKO participated in data collection and data management. SEM, MN, and KKO wrote the first draft of the manuscript. All co-authors reviewed and provided insightful comments to the analysis and manuscript drafts. All authors approved the final report. KKO is responsible for the overall content as the guarantor of this work.

Provenance and peer review: Commissioned; externally peer reviewed.

This article is part of a collection proposed by Swiss Re, which also provided funding for the collection, including open access fees. *The BMJ* commissioned, peer reviewed, edited, and made the decision to publish. Nita Forouhi, Dariush Mozaffarian, and David Ludwig provided advice and guided the selection of topics. The lead editors for the collection were Navjoyt Ladher, Rachael Hinton, and Emma Veitch.
